# Dr. O’Flanagan and His Wonderful Cures (Poetical)

**Published:** 1874-06

**Authors:** Amanda T. Jones


Herald of Health for May.

### *DR. O'FLANAGAN AND HIS WONDERFUL CURES.*

BY AMANDA T. JONES.

I'm Barney O'Flanagan, lately from Cork;
A sakin for practice I've coom to New York;
It's a dochter I am, and well versed in the thrade,
Who can mix yeez a powdher as good as is made.
Have yeez pains in yer bones, or a botherin' ache
In yer jints afther dancin' a jig at a wake?
Have yeez caught a black eye from some lounderin' whack?
Have yeez vertebral twists in the shpine av yer back?
Whin yer walkin' the strates are yeez likely to fall?
Don't whisky sit well on yer stomach at all?
Sure its botherin' nonsense to sit down and wape,
Whin a bit av a powdher 'ill put yeez to slape.
Shtate yer symptoms, me darlins, and niver yeez doubt,
But as sure as a gun I can shtraighten yeez out.

Thin don't yeez be gravin' no more;
Jist quit all your sighin' forlorn;
Och, Dochtor O'Flanagan's thrue to the core,
And what's more, he's a gintleman born!

Coom thin, ye poor craythurs, and don't yeez be scairt!
Have yeez batin' and lumberin' thumps at the hairt?
Wid ossification and accelleration,
Wid attenuation and regurgitation,

Wid amaciation and axacerbation,
Wid expectoration and wake cerculation,
Wid pracipitation and hapitazation,
Wid praoccupation and avaporation,
Wid hallycination and acrid sacration,
Wid black aruptation and putrification,
Wid great jactitation and cowagulation,
Wid quare titillation and cowld perspiration?
Be me sowl! but I'll bring all yer woes to complation;
Onless yer in love—*thin* ye're past all salvation.

Och, don't yeez be gravin' no more!
Jist quit all yer sighin' forlorn;
Ould Barney O'Flanagan's thrue to the core,
And indade he's a gintleman born!

'Sure, me friends, its the wonderful luck I have had.
In the tratement of sickness, no matther how bad;
All the hundreds I've cured its not aisy to shpake;
And if any sowl dies, fath I'm in at the wake!
There was Misthress O'Toole was tuck down mighty quare;
That wild there was niver a one dared to lave her.
And what was the matther? ye'll like for to hare—
'Twas the double quotidian humerous faver.
Well, I tuck out me lancet and pricked at a vein;
Och, murther! but didn't she howl at the pain!
Six quarts, not a dhrap less, I tuck, widout sham;
And troth, she shtopped howlin' and lay like a lamb.
Thin for fare sich a method of tratement was risky,
I hasthened to fill up its place wid ould whisky.

Thin don't yeez be gravin' no more;
Jist quit all yer sighin' forlorn;
Remimber O'Flanagan's thrue to the core,
And ye'll prove him a gintleman born.

Well, Misthress O'Toole was tuck better at once;
For she riz up in bed and cried "Paddy, ye dunce,
Give the dochter a dram." So I sat at me aise
And powerd down the punch jist as fine as ye plaze.
Thin I left a prascription all written down nate,
Wid ametics and diaphoretics complate,
Wid antispasmodics to kape her so quiet,
And a toddy so shtiff that ye'd all like to thry it.
So Paddy O'Toole mixed 'em well in a cup,
All barrin' the toddy—and that he dhrank up;
For he shwore 'twas a shame sich good brandy to waste
On a double quotidian faverish taste.
And fath we agrade it was not bad to take,
Whin we dhrank that same toddy nixt night at the wake.

Thin don't yeez be gravin' no more;
Jist quit all yer sighin' forlorn;

Whin ye thry Dochter Barney yer throubles are o'er,
Wid the help of a gintleman born.

There was Michael McDonegan down wid a fit,
Caught of dhrinkin' could wattther whin tipsy a bit.
'Twould have done yer heart good to have heard him cry out
For a cup of pottheen or a tankard of shtout;
Or a wee dhrap of usquebaugh fresh from the shtill—
And the snakes that he saw, troth 'twas jist fit to kill.
It was mania potatororum, bedad!
Howly Mither of Moses, the divils he had!
Thin to scare 'em away we surroonded his bed,
Clapped on forty laches and blisthered his head,
Bate all tin the pans, and set up sich a howl,
That the last fiery divil ran off, be me sowl!
And we writ on his tombstone: "He died av a shpell
Caught of dhrinkin' cowld watther shtrait out av a well."

Thin don't yeez be gravin' no more;
Arrah! quit all yer sighin' forlorn;
There's shtrychnine and vitril and whisky galore,
And the dochter's a gintleman born.

There was swate Ellen Mulligan sazed wid a cough,
And ivery one said it would carry her off.
"Whisht!" says I, "Thrust to me now, and don't yeez go crazy,
If the girlie *must* die, sure I'll make her die aisy."
So I sarched through me books for the thrue diathasis
Of morbus dyscrasia tuberculous phthasis;
And I boulsthered her up wid the strongest of tonics,
Wid iron and copper, and hosts of carbonics;
Wid whisky sarved straight in the finest av shtyle;
And I grased all her inside wid cod-liver ile;
And says she, whin she died: "Och Dochtor, me honey!
Its you as can give us the worth av our money;
And begorra! I'll shpake to the divil this day,
Not to kape yeez a waitin' too long for yer pay."

Thin don't yeez be gravin' no more;
Jist quit all yer sighin' forlorn;
Me patients are proud av me medical lore,
All because I'm a gintleman born.

There was Teddy Maloney, who bled at the nose
Afther blowin the fife; and mayhap ye'd suppose
'Twas no matther at all; but the books all agrade
'Twas a sarious visceral throuble indade.
Wid the blood shwimmin' round in a circle elliptic,
The Schneidarian membrane was wantin' a shtyptic;
The antarior nares were nadin' a plug,
And troth Ted himself was in nade of a jug.

Thin I rowled out a big pill of sugar of lead,
And I dosed him and shtood him up firm on his head;
And, says I, "Now me lad, don't be atin' yer lingth,
But dhrink all ye plaze, jist to kape up yer stringth."
(Och, his widdy's a jewel! but whist, don't ye shpake!
She'll be Misthress O'Flanagan airly nixt wake!)

Thin don't yeez be gravin' no more:
Jist quit all yer sighin' forlorn;
For yer widdies, belike, whin their mournin' is o'er,
Will marry some gintleman born.

Ould Biddy O'Cardigan lived all alone,
And she felt mighty nate wid a house av her own;
Shwate-smellin' and whoulsome, swaped clane wid a rake,
Wid two or thray pigs, jist for company's sake.
Well, what should she do but be tuck sick so vile,
Wid choleraphobia vomitus bile.
And she sint shtraight for me. "Dochter Barney, me lad,"
Says she, "I'm in nade of av assistance, bedad!
Have yeez niver a powdher, or bit av a pill;
Me shtomach's a rowlin', jist make it kape shtill."
"I'm the bye can do that," says I: "Hould on a minit!
Here's me medicine chist; wid me calomel in it;
And I'll make yeez a bowle full av rid pepper tay,
So shtrong ye'll be thinkin' the divil's to pay!"

Thin don't yeez be gravin' no more;
Jist quit all yer sighin' forlorn;
Here's drugs be the handful, and pills be the score,
And to dale them a gintleman born.

Wid a gallon of rum, thin a flip I created,
Swate wid mustard and spice, and the poker I hated
Rid hot as a guinea jist out of the mint;
And, begorra! shtraight into her stomach it wint.
Och, never belave me, but didn't she roar!
I'd have kaped her alive wid a quart or two more;
And the thray little pigs, in that house av her own,
Wouldn't now be a shtarvin' and squalin' alone;
And that gossoon, her bye, the shpalpeen altogither,
Would niver have sworn that I murdhered his mither.
Sure, for sayin' that same but I sarved him a thrick,
Whin I met him by chance—wid a bit of a shtick.
Fath, I dochtored him well, till the cure I complated,
And, be jabers, there's *one* man alive that I've thrated!

Thin don't yeez be gravin' no more;
Jist quit all yer sighin' forlorn;
Jist knock, whin yer sick, at O'Flanagan's door,
And die for a gintleman born!

